# Buffalo Hospital of the Sisters of Charity

**Published:** 1849-05

**Authors:** 


*Buffalo Hospital of the Sisters of Charity.*—We are gratified to announce the passage of a bill by the Legislature of this State, granting an appropriation of *nine thousand dollars*, for the benefit of this most excellent charity. This timely and generous support will enable the Institution to extend its scope of charitable relief, and places it upon a permanent basis. It is to be at once enlarged by the erection of a new building. The present accommodations are not adequate for more than one hundred patients. The proposed additions, will nearly double its capacity. A public hospital has long been a great desideratum in this city; and, inasmuch as other efforts to provide for the necessities of the suffering sick have proved abortive, the community, and especially they who need the benefits of an Asylum in disease, are under infinite obligations to those by whose philanthropic exertions this charity has been established.

